# 395. Remdesivir Stewardship Through Criteria for Use Implementation Across a Large System is Associated with Enhanced Outcomes and Efficiency

**DOI:** 10.1093/ofid/ofad500.465

**Published:** 2023-11-27

**Authors:** Florian Daragjati, Reese Cosimi, Subhangi Ghosh, Ana Cristina Perez Moreno, Collin Miller, Erin Rice, Mohamad G Fakih

**Affiliations:** Ascension, Jacksonville, Florida; Ascension, Jacksonville, Florida; Ascension, Jacksonville, Florida; Ascension Health, Franklin, Wisconsin; Ascension Health, Franklin, Wisconsin; Ascension, Jacksonville, Florida; Ascension, Jacksonville, Florida

## Abstract

**Background:**

The National Institute of Health (NIH) COVID-19 Treatment Guidelines support use of remdesivir in hospitalized adults with mild-to-moderate COVID-19 infection for those at risk of progressing to severe COVID-19 and those who require oxygen supplementation, high-flow nasal cannula, or non-invasive ventilation. We implemented a standardized approach for the treatment of COVID-19 infection adopting the NIH criteria for use in a single system.

**Figure:**
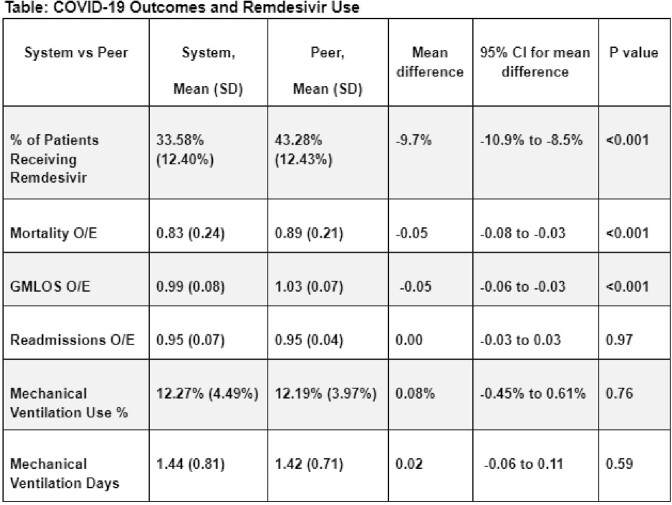

**Methods:**

Patients ≥ 18 years old admitted with a diagnosis of COVID-19 infection from January 2021 to September 2022 in 94 hospitals from a single health system and 1012 peer hospitals from a large national database were included. Risk-adjusted outcomes in COVID-19 patients were compared, including mortality, readmissions, geometric mean length-of-stay (GMLOS), and mechanical ventilation days. We also compared remdesivir use in the COVID-19 population in the single health system and the large national database.

**Results:**

85,874 and 789,660 COVID-19 patients were included from the single health system and large national database, respectively, during that time frame. Remdesivir use in COVID-19 patients was lower within the single system hospitals compared to external peer hospitals (33.6% vs 43.3% respectively; mean difference 9.7% (CI 8.5%-10.9%); p < 0.001). Risk-adjusted mortality and GMLOS were also lower for the system compared to peer hospitals, while readmissions and mechanical ventilation use were not significantly different (Table).

**Conclusion:**

The adoption of the NIH Criteria for remdesivir use in a single system was associated with lower COVID-19 mortality and lower geometric mean length of stay despite less remdesivir use compared to external peer hospitals. Our findings support optimizing the compliance with the NIH treatment guidelines.

**Disclosures:**

**Reese Cosimi, PharmD**, Allergen: Advisor/Consultant

